# Oxidative Stress during HIV Infection: Mechanisms and Consequences

**DOI:** 10.1155/2016/8910396

**Published:** 2016-10-13

**Authors:** Alexander V. Ivanov, Vladimir T. Valuev-Elliston, Olga N. Ivanova, Sergey N. Kochetkov, Elizaveta S. Starodubova, Birke Bartosch, Maria G. Isaguliants

**Affiliations:** ^1^Engelhardt Institute of Molecular Biology, Russian Academy of Sciences, Vavilov Str. 32, Moscow 119991, Russia; ^2^M. P. Chumakov Institute of Poliomyelitis and Viral Encephalitides, Moscow 142782, Russia; ^3^Cancer Research Center Lyon, INSERM U1052 and CNRS 5286, Lyon University, 69003 Lyon, France; ^4^DevWeCan Laboratories of Excellence Network (Labex), France; ^5^Riga Stradins University, Riga LV-1007, Latvia; ^6^Department of Microbiology, Tumor and Cell Biology, Karolinska Institutet, 17177 Stockholm, Sweden; ^7^N. F. Gamaleya Research Center of Epidemiology and Microbiology, Moscow 123098, Russia

## Abstract

It is generally acknowledged that reactive oxygen species (ROS) play crucial roles in a variety of natural processes in cells. If increased to levels which cannot be neutralized by the defense mechanisms, they damage biological molecules, alter their functions, and also act as signaling molecules thus generating a spectrum of pathologies. In this review, we summarize current data on oxidative stress markers associated with human immunodeficiency virus type-1 (HIV-1) infection, analyze mechanisms by which this virus triggers massive ROS production, and describe the status of various defense mechanisms of the infected host cell. In addition, we have scrutinized scarce data on the effect of ROS on HIV-1 replication. Finally, we present current state of knowledge on the redox alterations as crucial factors of HIV-1 pathogenicity, such as neurotoxicity and dementia, exhaustion of CD4^+^/CD8^+^ T-cells, predisposition to lung infections, and certain side effects of the antiretroviral therapy, and compare them to the pathologies associated with the nitrosative stress.

## 1. Introduction

Reactive oxygen species (ROS) is a general term of oxygen intermediates with high reactive capacity towards various biological molecules. They include hydroxyl radical (HO^•^), singlet oxygen (^1^O_2_), superoxide anion (O_2_^•−^), hydrogen peroxide (H_2_O_2_), and other reactive species [1, 2]. ROS are produced in various cellular processes and organelles: electron leakage from the mitochondrial electron transport chain (ETC), degradation of lipids, amino acids, and biogenic polyamines, protein folding in the lumen of endoplasmic reticulum (ER), and so forth [3–7]. The most reactive type of ROS is the hydroxyl radical. It is produced from hydrogen peroxide that oxidizes divalent iron cations via the Fenton reaction

(1) $$\begin{array}{c} {Fe}^{2+}+H_{2}O_{2}⟶{Fe}^{3+}+{HO}^{•}+{HO}^{-} \end{array}$$

or as a result of the Haber-Weiss cycle that involves a reduction of ferric ions by superoxide anions into ferrous ions followed by the Fenton reaction:

(2) $$\begin{array}{c} {Fe}^{3+}+{O_{2}}^{•-}⟶{Fe}^{2+}+O_{2} \end{array}$$

Thus, the net reaction of the Haber-Weiss cycle can be described as

(3) $$\begin{array}{c} {O_{2}}^{•-}+H_{2}O_{2}⟶{HO}^{•}+O_{2}+{HO}^{-} \end{array}$$

Superoxide anions have several sources in cells. First, they are generated in mitochondria. Electron transport through the ETC during oxidative phosphorylation is generally accompanied by escape of up to 1-2% of electrons that are trapped by molecular oxygen [7]. Alteration of mitochondrial bioenergetics by various factors usually gives rise to superoxide anion production. Secondly, superoxide anion is produced by a family of NADPH oxidases (NOX/DUOX), comprised of seven isoforms: NOX1–NOX5 and DUOX1-DUOX2 [6]. They transport electrons across the membranes and generate superoxide with the exception of NOX4 that produces hydrogen peroxide [8]. Activation of NOX-mediated ROS production can be achieved by various mechanisms. For example, NOX4 is controlled only at the level of transcription since this enzyme is constitutively active [6]. NOX1–NOX3 are generally induced on the transcriptional level and activated by a controlled assembly of the multisubunit complexes. Finally, several isoforms including NOX5 and DUOX1-DUOX2 possess calcium-binding domains that mediate additional level of ROS production. Third, superoxide anions are generated by cytochromes P450 (CYP) which catabolize various endogenous compounds and xenobiotics [9]. Hydrogen peroxide is mainly formed as a stoichiometric by-product in catabolic reactions and through formation of disulfide bonds during protein folding in the ER [5, 10]. Finally, reactive oxygen species can derive from the activity of xanthine oxidoreductase (XOR) [11, 12]. XOR is widely distributed throughout various organs including the liver, gut, lung, kidney, heart, and brain as well as the plasma. It is generally accepted that the enzyme is normally present* in vivo* as an NAD-dependent cytosolic dehydrogenase (XDH), incapable of ROS production. However, sulfhydryl oxidation or limited proteolysis converts the XDH into xanthine oxidase (XO) which produces superoxide and hydrogen peroxide, with the latter being the major product under physiological conditions [11]. Furthermore, both XO and XDH can oxidize NADH, with the concomitant formation of the reactive oxygen species [11, 12].

Different types of ROS are characterized by their varying ability to react with biological molecules. The most reactive ROS is the hydroxyl radical, HO^•^, the one-electron oxidized form of the hydroxide ion (HO^−^) [1, 13]. It can oxidize almost any molecule in its proximity including DNA, phospholipids, and proteins [13, 14]. Oxidation results in the accumulation of 8-oxoguanine (8-oxoG) and other oxidized nucleic bases, malondialdehyde (MDA), and 4-hydroxynonenal (HNE) as typical lipid peroxidation products and in protein damage manifested in the increase of the protein carbonyl content [15]. Much less active is the superoxide anion: its reactivity is hampered by a negative charge of the species; however, its protonation generates the perhydroxyl radical (HO_2_^•^) with a higher oxidizing potential [16]. The reaction potential of H_2_O_2_ is also very low; however, it is converted into the hydroxyl radical with a much higher oxidizing capacity [17]. H_2_O_2_ also possesses a unique (for ROS) capacity to cross biological membranes which turns it into a classical signaling molecule [17, 18].

Eukaryotic cells have developed multiple mechanisms of ROS neutralization (“scavenging”) in order to protect themselves against oxidation of biological molecules. First, ROS can be neutralized directly by the low molecular weight compounds referred to as antioxidants, such as vitamins C and E and glutathione (GSH) [19], and a wide set of ROS-converting enzymes [20] including NAD(P)H:quinone oxidoreductase 1 (Nqo1) that scavenges superoxide anion [21] and superoxide dismutases (SODs) that convert O_2_^•−^ into H_2_O_2_ [22]. SODs exist in three isoforms expressed in different cellular compartments: SOD1 (Cu/Zn-SOD) is mostly localized in the cytoplasm; SOD2, (MnSOD) in the mitochondrial matrix; and SOD3 (EC-SOD), at the cell surface. Neutralization of H_2_O_2_ is performed by multiple enzymes such as catalase (CAT), glutathione peroxidases (GPx, eight isoforms), and peroxiredoxins (Prdx, six isoforms) [23, 24]. Of these enzymes, GPx4 and 1-Cys peroxiredoxins are responsible for scavenging lipid peroxides thus protecting lipids from the oxidative damage [25–27]. Additional protection from ROS is mediated by heme oxygenase, the rate-limiting enzyme of heme catabolism which leads to the release of free iron, which in turn offers protection against oxidative stress [28]. Other antioxidant proteins include enzymes that mediate biosynthesis of glutathione and proteins that recycle oxidized glutathione, peroxiredoxins, and glutathione peroxidases (glutaredoxins and thioredoxins) [20, 29]. Noteworthily, expression of a wide set of antioxidant enzymes is controlled by NF-E2-related factor 2 (Nrf2), a transcription factor that recognizes a common short sequence, referred to as Antioxidant Response Elements (ARE), in the promoters of genes encoding ROS-converting enzymes [20]. Components of antioxidant defense systems differ in their capacity to neutralize ROS. Hydrogen peroxide is much more efficiently neutralized by peroxiredoxins and glutathione peroxidases, while classical antioxidants such as glutathione have a much lower potential [16, 30]. The actual levels of ROS are defined by the balance between the activities of ROS-generating and ROS-scavenging molecules, being different for different cellular compartments [31].

Several techniques are currently used to analyze the redox status of the cell and to determine the levels of ROS. Firstly, oxygen radicals can be detected by the electron paramagnetic resonance (EPR) using a spin-trapping technique; however, this method requires highly specialized equipment [32]. Secondly, ROS levels can be quantified indirectly using low molecular weight compounds (sensors) that are oxidized by ROS into fluorophores. They include 2′,7′-dichlorodihydrofluorescein (DCFH_2_DA), dihydroethidium (DHE) and its mitochondrially targeted derivative MitoSOX, and boronate probes [33, 34]. Protein sensors such as HyPER or roGFP can be introduced as genes which makes them suitable for measuring ROS levels in almost any organelle [35–37]. Thirdly, the oxidative stress can be assessed indirectly by evaluating the levels of oxidative stress biomarkers, such as stable (by-)products generated under conditions of oxidative stress which enter the tissues, cells, or circulation, such as oxidized glutathione (GSSG), MDA, and HNE (for lipids) and 8-oxoG (DNA) and protein carbonyls [15, 38]. In addition, the cellular redox state can be quantified by estimating the capacity of blood/serum/tissue samples to oxidize/reduce some standard compounds that mimic cellular targets of ROS (e.g., see [39, 40]).

Oxidative stress accompanies a wide variety of viral infections including those induced by hepatitis B (HBV) [41], C (HCV) [42], and delta (HDV) [43] viruses, herpes [44, 45], respiratory [46, 47], and other viruses. In this review, we will summarize data on the mechanisms by which HIV triggers massive ROS production in the host cell and deregulates the antioxidant defense system. We will also present current concepts on the role of HIV-induced oxidative stress in the development of HIV-associated pathologies.

## 2. HIV-1 Biology

The human immunodeficiency virus type-1 (HIV-1) is a lentivirus that infects and by various mechanisms kills vital cells of human immune system, such as T-helper cells, macrophages, and dendritic cells, thus causing immunodeficiency [48, 49]. The acquired immunodeficiency syndrome (AIDS) is a condition in humans in which the progressive failure of the immune system undermined by HIV-1 infection allows life-threatening opportunistic infections and cancers to thrive. Without treatment, the survival time of HIV-1 infected individuals is estimated to be 9 to 11 years. However, during the three decades since its discovery, 27 antiretroviral drugs have been approved for HIV therapy [50]. Current antiretroviral therapy (ART), based on combinations of 3-4 drugs, now allows us to efficiently suppress HIV viral load and to prolong life of HIV/AIDS patients almost to the one of the general population, at least in high-income countries [51].

HIV is a single-stranded, positive-sense, enveloped RNA virus. The genome carries nine genes (*gag*,* pol*,* env*,* tat*,* rev*,* nef*,* vif*,* vpr*, and* vpu*) that encode 19 proteins; the coding sequence is framed by the long terminal repeats (LTRs) [48, 49]. Three of these genes,* gag, pol,* and* env*, contain information needed to make new viral particles. Processing of* pol* gene results in formation of three enzymes: reverse transcriptase (RT), integrase, and protease. Translation of* env* gene produces glycoprotein 160 (Gp160) that further is processed to give Gp120 and Gp41.* Gag* gene ensures production of matrix (MA), capsid (CA), nucleocapsid (NC), and P6 proteins as well as spacer peptides 1 (SP1) and 2 (SP2). The six remaining genes,* tat*,* rev*,* nef*,* vif*,* vpr*, and* vpu* (or* vpx* in the case of HIV-2), are regulatory genes for proteins that control the ability of HIV to infect cells, produce new copies of virus (replicate), or cause the disease [52]. Upon entry into the target cell, HIV reverse-transcribes the RNA genome into the double-stranded DNA, transports it into the cell nucleus, and integrates into the chromosomes, the activities mediated by virus-encoded enzymes reverse transcriptase and integrase, and cellular cofactors [48]. Once integrated, the virus may become latent, which allows the infected cells to avoid detection by the immune system. Alternatively, the virus may be transcribed and translated, producing new RNA genomes and viral proteins that are packaged and released from the cell as new virus particles to start the new infection cycle.

## 3. Oxidative Stress during HIV Infection

To date, numerous lines of evidence show that HIV infection triggers pronounced oxidative stress in both laboratory models and the context of* in vivo* infection. HIV-infected individuals exhibit enhanced ROS production in monocytes [53] and severely elevated levels of oxidized nucleic bases such as 8-oxoG and lipid peroxidation products, including MDA in plasma and alkanes in the breath output [54–63].

Compensation of the pathogenic effects of HIV-1 replication requires intact functions of ROS detoxifying enzymes. Parsons et al. showed that HIV-1 individuals with a null-allele polymorphism in* gstm1* gene, associated with a loss of function of the Phase II detoxifying enzyme glutathione S-transferase [64], exhibit lower count of CD4 T-cells, increased HIV viral load, and increased 8-oxoG in mitochondrial DNA [65]. However, HIV-infected individuals demonstrate a reduction of total antioxidant capacity [59], decreased GSH/GSSG ratio in epithelial lung fluid [3], and decreased GSH content in blood [56, 58, 61, 63, 66–69]. Marked elevation of ROS levels was also detected in the HIV-infected cell cultures [70, 71]. The most profound decrease of the total antioxidant capacity was detected in subsets of CD4^+^ and CD8^+^ T-lymphocytes [66], with low CD4 T-cell counts correlating with more severe oxidative stress [59, 60, 62, 72]. Corroborating these observations, the number of CD4^+^ cells positively correlates with the total levels of ROS scavengers such as glutathione [56]. Noteworthily, these changes are more pronounced in treatment-naive patients than in patients under ART [58, 61] since ART restores the numbers of CD4^+^ T-cells but at the same time augments the imbalance of the redox status [73]. In HIV/HCV-coinfected patients, the levels of oxidative stress markers are generally higher than in individuals with HIV monoinfection, as indicated by MDA and GSSG plasma levels [74, 75].

Elevated levels of the oxidative stress markers are also detected in other tissues and body fluids. Brain tissues (brain frontal cortex collected from autopsia) of HIV-infected individuals are characterized by the increased levels of 8-oxoG in the nuclear DNA [76] and increased HNE levels [69]. Elevated levels of superoxide radical and HNE are also detected in the cerebrospinal fluid [69, 76]. Interestingly, similar effects are observed in the NL4-3Δ transgenic rat model expressing HIV proteome devoid of the Gag-Pol polypeptide. In these animals, high levels of superoxide anion can be indirectly detected by electron spin resonance spectroscopy/ESR using CMH probe in the aortas [77] and by fluorescent microscopy with DHE dye in the lungs [78]. Altogether, this indicates that HIV-1 actively interferes with the development of oxidative stress response.

## 4. Mechanisms of ROS Production during HIV Infection

HIV-1 induces oxidative stress by deregulation of oxidative stress pathways with escalation of ROS production and by inducing mitochondrial dysfunction [70, 71]. The enhancement of ROS production is mediated by the envelope protein Gp120 [79–85], Tat [83, 84, 86–88], Nef [89–91], Vpr [71, 92, 93], and reverse transcriptase (RT) [94].

**Figure 1:**
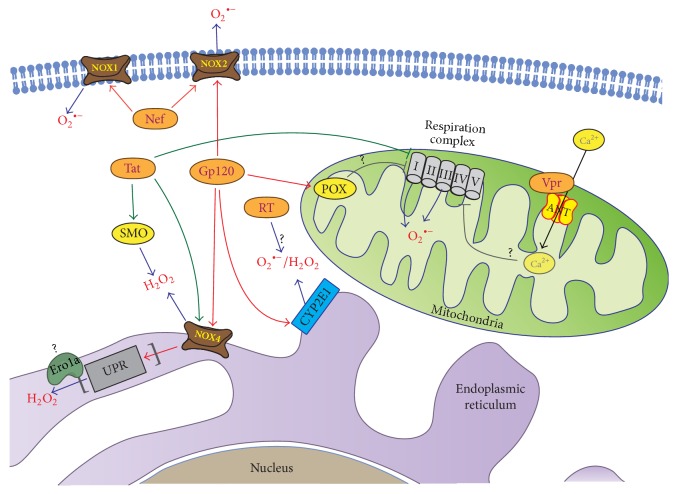
Cellular sources of reactive oxygen species in HIV infection. Several HIV proteins enhance ROS production by different mechanisms. These viral proteins include amongst others the envelope protein Gp120, Tat, Nef, Vpr, and RT. The envelope protein Gp120 enhances ROS production via upregulation of cytochrome P450 2E1 (CYP2E1), proline oxidase (POX), and activation of NOX2 and NOX4. Tat protein induces spermine oxidase (SMO), an enzyme involved in catabolism of biogenic polyamines, and may impact mitochondrial function. Tat also activates NADPH (but not xanthine) oxidases and in particular Nox4, which in turn may induce other peroxide-generating enzymes involved in unfolded protein response (UPR) such as ER oxidoreductin 1*α* (Ero1*α*). Vpr protein interacts with adenine nucleotide translocator (ANT, a component of mitochondrial permeability transition pore (PTP)) that is implicated in Ca^2+^ influx into mitochondria. Nef protein can directly interact with the p22phox subunit of NADPH oxidases without affecting NOX expression. Finally, RT triggers ROS production by yet undiscovered mechanism(s).

The envelope protein Gp120 enhances ROS production in various cell lines of lymphoid origin [82], in endothelial brain cells [83], microglia cells, neurons, and astrocytes [79, 80]. In astrocytes, it enhances ROS production by several parallel mechanisms: via cytochrome P450 2E1 (CYP2E1), NOX2 and NOX4, and the Fenton-Weiss-Haber reaction (Figure 1) [79, 95]. The effect of Gp120 on CYP2E1 is mediated through upregulation of CYP2E1 expression. Interestingly, however, EPR analysis of the HIV-1 infected monocyte-derived macrophages revealed no increase in the production of either hydroxyl or other oxygen radicals [96]. In neuroblastoma cells, Gp120 was shown to induce proline oxidase (POX) that produces pyrroline-5-carboxylate with a concomitant generation of ROS (Figure 1) [85].

The regulatory Tat protein triggers ROS production via several independent mechanisms (Figure 1). The first involves the NADPH (but not xanthine) oxidases [86]. The second implies the induction of spermine oxidase (SMO), an enzyme involved in the catabolism of biogenic polyamines [88, 97]. The third relies on mitochondrial dysfunction [98] but was questioned in a later study [86]. A detailed analysis of the levels of ROS in different subcellular compartments of the HIV-1 infected cells revealed no significant increase in the content of H_2_O_2 _in either cytoplasm or mitochondria but a strong increase in the ER [99]. ER is the primary “residence” for NOX4 that produces hydrogen peroxide [8, 100]. An increase in H_2_O_2_ levels in ER of HIV-1 infected cells was demonstrated using a genetically encoded ratiometric HyPER sensor [99]. Moreover, in these cells, NOX4 mediated the induction of unfolded protein response (UPR). In concordance with these data, an elegant study demonstrated that the suppression of NOX4 by RNAi in Tat-expressing cells results in a significant reduction of H_2_O_2_ levels in the ER [99]. The results generated using HyPER sensor could be questioned. Such sensors have been used in a number of studies (such as [4, 99]) that demonstrated that the dynamic range of its signal is small [101] to negligible [102], with changes in the HyPER_ER_ fluorescence reflecting not so much the changes in peroxide levels but rather the influence of other factors such as proline disulphide isomerases (PDI) [103]. Also, one cannot rule out that NOX4 contributes to the induction of oxidative stress indirectly, through the induction of other peroxide-generating enzymes involved in UPR. If so, one could propose a component of the protein-folding machinery which could be involved, namely, ER oxidoreductin 1*α* (Ero1*α*) [104] which is upregulated within the PERK branch of UPR (Figure 1) [105]. Intriguingly, the elevation of hydrogen peroxide levels in the ER contradicts the existing concept on the efficient neutralization of hydrogen peroxide in the ER by scavenging enzymes, including peroxiredoxin 4 [106] and glutathione peroxidases 7/8 [101, 107]. This may not be widely accepted, which leaves open the actual mechanism of the Tat-mediated oxidative stress in the ER.

HIV-1 Nef protein has shown a prooxidant activity in microglial cells and in neutrophils [89–91]. The activity is related to the ability of Nef to interact with Vav protein (Figure 1) [89]. Vav is a nucleotide exchange factor for Rac1 that is recruited to the NOX1–NOX3 complexes [6], with the p22^phox^ subunit of NADPH oxidases, but without affecting NOX expression [91]. These interactions are in perfect concordance with the absence of changes in the expression of NOX1, NOX2, and NOX4 in NL4-3Δ* gag-pol* transgenic rats compared to the wild-type animals [77].

Viral protein R (Vpr) is another important regulator of ROS production [108]. In yeast, Vpr expression induces an oxidative stress leading first to the decreased levels of superoxide anion and hydroxyl radical as well as glutathione and significantly decreased activities of catalase, glutathione peroxidase, glutathione reductase, glucose-6-phosphate dehydrogenase, and glutathione S-transferase and later on to elevated levels of superoxide anion and peroxides and increased activities of most of antioxidant enzymes [108]. It was shown that Vpr triggers oxidative stress by causing mitochondrial dysfunction [92, 109, 110] and ROS production in mitochondria (Figure 1) [71]. Mitochondrial dysfunction is promoted by binding of Vpr to the adenine nucleotide translocase (ANT) [110], a protein that forms an inner channel of the mitochondria permeability transition pore (PTP) [110]. This indicates the propensity of Vpr to unbalance the redox state of the cells contributing to the HIV-1 pathology.

Mitochondrial dysfunction is a general mechanism of ROS production common for most viral infections [111–113]. NADPH oxidases and CYP2E1 serve as the major sources of ROS in infections with human hepatitis C, influenza, and respiratory syncytial viruses [114–121]. The overview of the field demonstrates that sources of ROS operational in HIV-1 infection follow similar trends.

## 5. HIV and Antioxidant Defense Pathways

The effect of HIV-1/HIV-1 proteins on the cellular antioxidant defense system is debatable. Several groups reported a decrease in SOD (SOD3 in particular), CAT, and GPx activities in plasma of the HIV-infected individuals [61, 63, 75, 122]. The data on Gp120 is controversial; it was shown to either enhance [123] or not affect the expression of* sod2* gene [82]. However, the individual Tat protein causes an opposite effect: it suppresses the expression of MnSOD through inhibition of binding of Sp1 and Sp3 transcription factors to* sod2* gene promoter and binding to its mRNA [124, 125]. In addition, studies done in HIV-1 NL4-3Δ transgenic rats demonstrate a decrease in the Cu/Zn-SOD expression, whereas the expression of MnSOD remains unaltered [77].

Overall, both Gp120 and Tat suppress expression of the glutathione synthesizing and metabolizing enzymes. Both downregulate the expression of glutathione synthase (GSS), glutathione reductase (GR), and GPx, leading to a decrease in the total glutathione content and an increase of the GSSG/GSH ratio [83, 84, 123, 126]. Gp120 also shows a strong ROS-dependent inhibitory effect on the expression of glutamine synthase (GS) [127]. Interestingly, Tat exhibits a stronger inhibitory effect on glutathione than Gp120 [83]. In addition to the inhibition of GSH biosynthesis pathways, Tat induces the expression of glutathione peroxidase isoform GPx4 [126], which scavenges lipid peroxides. At the same time, Tat has no effect on the expression of thioredoxin reductase [126], an enzyme that reduces thioredoxin, which in turn reduces glutathione peroxidases and peroxiredoxins [29]. Vpr is yet another virus protein that triggers a decrease in the GSH levels [128]. The latter is caused by the suppression of ATP biosynthesis in mitochondria [128] (two molecules of ATP are required for biosynthesis of every glutathione moiety [129]).

A majority of glutathione metabolizing genes are controlled by the Nrf2 transcription factor [20].* In vivo*, HIV-1 appears to suppress the Nrf2/ARE pathway. Indeed, brain cortex tissues of HIV-1 infected individuals demonstrate the decreased levels of heme oxygenase 1 [130]. This effect is not mediated by Tat, Nef, or Vpr proteins but is apparently due to the replication of the viral genome. HO-1 protein expression correlates negatively with HIV replication levels.* In vitro* analysis of HO-1 expression in HIV-infected macrophages, a primary central nervous system (CNS) HIV reservoir along with microglia, demonstrated a decrease in HO-1 as HIV replication increased; HO-1 repression was mediated by high levels of IFN-*γ* concomitant with virus replication in the CNS [131]. While HIV replication seems to (indirectly) suppress the Nrf2/ARE pathway, the effects of the individual viral proteins are the opposite. HIV reverse transcriptase activates Nrf2 and upregulates the transcription of both HO-1 and Nqo1, at least in the cell culture system [94]. An ability to activate the Nrf2/ARE pathway was recently reported also for Tat [88]. It is mediated through the induction of spermine oxidase and concomitant production of hydrogen peroxide. Gp120 induces yet another classical Nrf2-dependent gene, multidrug resistant protein 1 (Mrp1) [81]. Such discrepancy between the factual data from* in vitro* studies and the status of Nrf2/ARE signaling during HIV infection has been observed also for other viruses such as HCV [132–135]. The actual (also long-term) effects of HIV-1 on the Nrf2/ARE pathway and their outcomes for the pathogenesis of HIV-1 infection remain to be elucidated.

## 6. ROS in HIV's Life Cycle

Hypoxia induces oxidative stress via an overgeneration of ROS [136]. A crucial role in the mammalian response to oxygen levels is played by the transcription factor Hypoxia-Inducible Factor-1 (HIF-1). Increased expression of HIF-1*α* contributes to the mitochondrial activity and ROS formation during the hypoxia [137]. HIV-1 protein Vpr induces HIF-1 resulting in the ROS-dependent activation of HIV LTR [71]. HIV-1 LTR is activated even by low concentrations of H_2_O_2_ [138] (whereas antioxidants inhibit viral transcription [139]). Further enhancement of the transcription is triggered by proinflammatory cytokines, including TNF-*α* [140, 141] induced through the redox-dependent NF-*κ*B pathway [142]. Additional influence of elevated ROS levels on HIV life cycle is achieved through the redox-sensitive transcription factors AP-1 and p53 [143]. Interestingly, transcription activation by exogenous hydrogen peroxide takes place only after the prolonged treatment, allowing us to hypothesize that virus-induced oxidative stress can play a crucial role in activation of the latent viral infection [138]. Supporting this, activation of the latent infection was triggered by modest changes in the cell redox potential (25 mV) [144]. Such changes can be induced either directly by HIV proteins or indirectly through the induction of proinflammatory cytokines such as TNF-*α* [145].

Oxidative stress may be also beneficial for the late stages of the HIV life cycle, since glutathione treatment of chronically infected cells leads to the abrogation of virion budding and release [146] preventing the infection of new T-cells [147]. The addition of GSH or GSH analogues is able to block late steps of viral replication [148], possibly by inhibiting the proper folding of glycosylated surface viral proteins in the ER, as was demonstrated for influenza virus [149]. The inhibitory effects of antioxidant treatment could also be attributed to the ability of ROS to induce CXCR4 receptor [150] as well as the glucose transporter Glut1 [151]. Enhanced expression of Glut1 was observed in both the infected cell cultures [152] and the neuronal tissues of the patients [153]. It leads to the elevation of glucose influx into the lymphocytes, monocytes, and epithelial cells. Enhanced glucose flux is known to promote infection with oncogenic viruses [154, 155]. In case of HIV-1, it also leads to augmented ROS production and enhanced infection of target cells [147, 156, 157]. However, opposite data were also reported: overexpression of peroxide-scavenging enzyme, GPx1, enhances production of HIV virions, whereas treatment of such cells with buthionine sulfoximine (BSO) that inhibits glutathione biosynthesis inhibits such increase [158].

## 7. ROS in HIV-1 Related Pathologies

HIV-induced oxidative stress plays an important role in the development of a wide spectrum of virus-associated pathologies. Among them are neurotoxicity and dementia and immune imbalance with the exhaustion of the pool of CD4 T-lymphocytes, as well as lung and cardiovascular disorders.

### 7.1. CNS Toxicity

**Figure 2:**
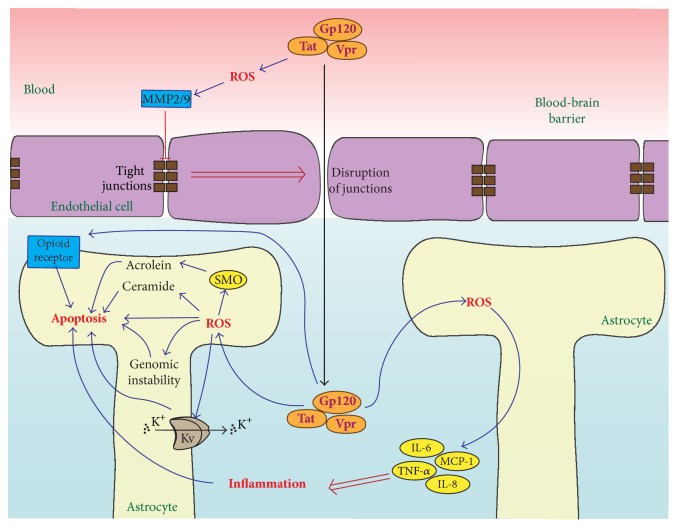
Mechanisms of HIV neurotoxicity. Enhanced ROS production, triggered by gp120, Tat, and Vpr proteins that circulate in the blood, results in alteration of blood-brain barrier (BBB) through matrix metalloproteinase 2/9- (MMP2/9-) mediated disruption of tight junction receptors ZO-1, laminin, claudin 5, and occludin. Gp120, Tat, and Vpr proteins activate a consequence of proapoptotic events. They include (i) oxidation of DNA and consequent genomic and mitochondrial DNA instability, (ii) increased lipid peroxidation and accumulation of ceramide that aggravates toxicity, (iii) induction of spermine oxidase (SMO) augmenting oxidative stress and producing toxic acrolein, (iv) stimulation of A-type transient outward K^+^ currents by Kv channels, and (v) induction of proinflammatory cytokines. In addition, it upregulates expression of opioid receptors that contribute to neurotoxicity in HIV-infected drug addicts.

Neurotoxicity and dementia are believed to be the direct consequences of HIV-1 infection: a majority of the cases with these neurological symptoms below 60 years of age are AIDS patients. HIV-1 affects the microglial cells; progressive infection leads to damage to astrocytes and neurons [159]. Levels of oxidative stress markers such as mitochondrial 8-oxoG in serum inversely correlate with the volume of the grey substance from selected brain areas (hippocampus, pallidum, etc.) [160]. Moreover, an increase in 8-oxoG in the nuclear DNA is accompanied by a decrease in the mitochondrial DNA content observed in the frontal cortex of the patients, altogether pointing at a direct link between ROS and neurological pathologies in AIDS patients [160]. The accumulated data points at the neurotoxicity being triggered by Gp120, Tat, and Vpr proteins which can penetrate the blood-brain barrier (BBB) (Figure 2) [161, 162]. Penetration is likely due to the disruption of BBB through several redox-regulated processes, including the induction of matrix metalloproteinases (MMP) 2 and 9 that target BBB tight junction receptors ZO-1, laminin, claudin 5, and occludin ([84, 163, 164], see also a comprehensive review by Toborek et al. [165]).

Gp120, Tat, and Vpr proteins contribute to the CNS pathology by both direct and indirect mechanisms (Figure 2). The direct mechanism involves induction of ROS production, which leads to the exhaustion of the antioxidant defense system and decreased cell viability [79, 98, 128, 166, 167]. Elevated levels of ROS result in the enhanced oxidation of DNA nucleic bases in both the nucleus and mitochondria, while their removal and DNA reparation are inhibited through suppression of DNA glycosylase 1 (enzyme the function of which is the removal of 8-oxoG; OGG1) [76]. This scenario leads to DNA instability, particularly to the deletion of the D-loop in mitochondrial DNA. Significantly, contribution to neurotoxicity of Gp120 and Tat is made by an increased lipid peroxidation and accumulation of ceramide [69].

An indirect promotion of CNS pathology is believed to be mediated by the enhanced production of the inflammatory cytokines and chemokines in astrocytes and microglia [159]. Gp120 and Vpr induce TNF-*α*, IL-6, IL-8, and MCP-1 in the ROS-dependent fashion (Figure 2) [80, 87, 167, 168]. In addition, Gp120 stimulates A-type transient outward K^+^ currents that contribute to the cell death [169]. Notably, this effect is also ROS-dependent [80]. An additional contribution to the pathogenic effects could come from the induction of spermine oxidase, an enzyme that mediates one of the two alternative pathways of polyamine metabolism [5]. It catalyses a reaction that yields H_2_O_2_ and acrolein as stoichiometric by-products. The latter compound is implicated in the brain pathology during ischemia-reperfusion [170, 171]. The induction of SMO may therefore represent an important mechanism of the HIV-induced brain damage. Finally, recent data of Pandhare et al. revealed that Gp120-mediated induction of proline oxidase leads to autophagy that at least partially alleviates neurotoxicity [85].

Interestingly, certain regions of AIDS patient brain are characterized by an increased expression of the opioid receptors [172]. In line with this, drugs such as morphine and amphetamine can per se trigger ROS production and dysregulate the antioxidant defense system, augmenting the pathogenic properties of Gp120 (Figure 2) [79, 123]. This may account for a more severe progression of the disease in the intravenous drug users.

### 7.2. Redox Associated Cardiovascular and Lung Pathologies

HIV-1 infection is accompanied by an increased risk of various cardiovascular diseases including arterial hypertension [173], atherosclerosis [174, 175], injury to coronary arteries [176], vasculitis [177], pericarditis, and myocarditis [173]. HIV-associated lung pathologies include increased susceptibility to infections, emphysema, and lung cancer [178, 179]. Their development is believed to be promoted by virus-induced oxidative stress. Oxidative stress in the lung leads to a decreased expression of the tight junction receptors, disrupting the epithelium and rendering lungs more susceptible to the microbes [180]. Moreover, treatment with lipopolysaccharide aggravates the redox imbalance in HIV-infected cells [78]. It may be speculated that these redox perturbations can trigger the inflammatory response, resulting in the tissue damage, as well as causing the genomic instability.

### 7.3. Effects of the Oxidative Stress on the Immune System

Very recent vivid example of the effects of oxidative stress on retroviral infection was provided by the study of Brundu et al. in a murine model [181]. Infection with the murine leukemia virus LP-BM5 causes murine AIDS, a disease characterized by many dysfunctions of the immunocompetent cells. Mice infected with LP-BM5 murine leukemia have a marked redox imbalance reflected by GSH and/or cysteine depletion in multiple immune organs/tissues. Significant decrease in cysteine and GSH levels was measured also in pancreas and in the brain, respectively [181]. Mice demonstrated a predominance of T-helper 2 (Th2) responses manifested by the expression of Th2 cytokines. Their peritoneal macrophages expressed the genetic markers of the alternative M2 macrophage polarization as Fizz1, Ym1, and Arginase 1 [181]. Conversely, macrophages capable of expressing iNOS (a marker of classical activation of macrophages) produced predominantly T-helper 1 (Th1) cytokines [181]. Restoration of the GSH/cysteine levels in the infected mouse organs (done with a N-acetyl-cysteine supplier) reduced the expression of M2 macrophage markers and increased the production of IFN-*γ*, while decreasing the production of Th2-cytokines as IL-4 and IL-5 [181]. Interestingly, this is not the first report of the association between the Th2 polarization and alteration of the redox status by retroviral infection and/or retroviral proteins. We have earlier shown that HIV-1 reverse transcriptase induces potent oxidative stress and, when expressed in mice as DNA immunogen, induces potent strongly Th2-polarized type of specific immune response [94]. Thus, HIV-1 infection and even expression of HIV-1 antigens induce an immune imbalance marked by M2-shift of the macrophage response and Th2-shift of the T-cell profiles which together promote the continuation of viral replication.

A separate set of immune abnormalities in HIV-1 infection is linked to the abnormalities in the tryptophan metabolism. In HIV-1 infected individuals, these abnormalities correlate with the enhanced oxidative kynurenine pathway of tryptophan catabolism [182, 183]. This pathway generates quinolinic acid, 3-hydroxykynurenine, and 3-hydroxyanthranilic acid, all of which are known to have the ability to generate free radicals [184]. Indoleamine 2,3-dioxygenase (IDO) is an intracellular enzyme involved in the first step of tryptophan catabolism [185]. Increased IDO expression occurs during human [186] and simian [187] retroviral infections. The data on the murine LP-BM5 immunodeficiency-causing retroviral infection is contradictory [188, 189]. In HIV-1 infection, increased IDO mRNA correlates with increased viral loads, while ART decreases IDO expression, which may be anticipated as a proof of the direct correlation between IDO and HIV virus propagation [190].

Catabolism of tryptophan by IDO leads to the reduction in tryptophan levels [191]. Th1 cell clones are more sensitive to changes in tryptophan levels than Th2 cell clones, resulting in a selective immunosuppression with the shift of the immune response towards the Th2-type [192]. Besides, IDO activity results in the increased levels of toxic downstream metabolites and generation of free radicals which contribute to Th1-cell suppression [191]. Furthermore, IDO activity causes even more imbalance in the T-cell subsets by increasing the proportion of T-regulatory [193] and decreasing the proportion of T-helper 17 cells [194]. In chronically infected hosts, the dysregulated activation/alterations in the immune regulatory mechanisms involving IDO lead to a compromised antiviral response and enhancement of viral replication [195–197]. In short, chronic IDO activation leads to the immune impairment, whereas IDO inhibition represses viral replication. Thus, the effects of IDO on the viral replication may in fact be indirect, being modulated by the disturbances in the virus-specific immune response.

These series of studies demonstrate that longitudinal (chronic) oxidative stress has detrimental consequences to the HIV-1 specific immune response, impairing the capacity of the body to control viral replication. On the contrary, suppression of the chronic oxidative stress with restoration of the antioxidant levels can reestablish the disturbed Th1/Th2 balance and open a possibility to control retroviral infection.

### 7.4. T-Cell Exhaustion

ROS levels correlate inversely with the CD4^+^ cell counts [59, 60, 62, 72, 198]. This may relate to a decrease in the reduced glutathione pool and the exhaustion of ROS-scavenging systems of the host cells [68, 70, 199]. It could also be due to the accumulation of DNA damage in these cells due to both increased production of ROS and the suppression of the respective DNA repair enzymes [62]. The molecular interrelations between HIV-induced oxidative stress and CD4^+^/CD8^+^ cell exhaustion remain to be investigated.

### 7.5. Pathological Consequences of the Nitrosative Stress

HIV is capable of infiltrating the brain and infecting brain cells. In the years following HIV infection, patients show signs of various levels of neurocognitive problems termed HIV-associated neurocognitive disorders (HAND) which afflict about half of HIV-infected patients. In Section 7.1, we described multiple links between neurological pathologies in AIDS patients and ROS. It is important to note that the latter are attributed not only to the oxidative but also to nitrosative stress and overproduction of nitrosative species during neuroinflammation [200]. Both processes occur due to the early direct and indirect effects of the viral proteins and through the late effects on mitochondrial integrity during apoptosis. There is clear experimental and clinical evidence linking the CNS symptoms of HIV with the effects of reactive nitrogen species (RNS), specifically nitric oxide (NO).

Mammalian cells generate NO as a by-product of NO synthase (NOS) activity. Neurons express neuronal NOS (nNOS), a constitutive isoform that synthesizes moderate amounts of NO; glial cells express inducible NOS (iNOS), which generates major NO amounts [201]. The nitrosative species are involved in the posttranslational modification of the brain proteome. NO is required for regular neuronal function, is produced by neuronal (nNOS), endothelial (eNOS), and inducible (iNOS) nitric oxide synthases, and is an important neurotransmitter in the brain.

At the same time, NO is the main mediator of mitochondrial dysfunction associated with HIV central nervous system symptoms, with an increased production of NO related to HIV-associated dementia. Nitrosative stress in microglia and astrocytes can be promoted by the individual viral proteins, such as Tat [202, 203]. Another viral protein Gp41 (its N-terminus) induces iNOS protein activity [204]. HIV-1 Gp120 is also involved in the induction of iNOS leading to the nitrosative stress [205]. Recent study by Mangino et al. in the murine model suggests a potential role in the promotion of the neuronal injury of the extracellular Nef which upregulates the expression of iNOS and production of NO [206].

The data on the effects of RNS outside of the brain is less “homogenous.” The overproduction of NO and the reduction of mitochondrial transmembrane potential correlate with the level of apoptosis in PBMCs of HIV-1 patient [200, 207]. This may be explained by the inhibitory effects of NO on the electron transport chain in the mitochondria as well as by the amino acid modifications. Amino acid modifications ascribed to NO are associated with the S-nitrosylation of cysteine and nitration of tyrosine and tryptophan (resulting in 6-nitro tryptophan or nitrohydroxytryptophan). The latter may be, at least in part, responsible for the abnormalities in the tryptophan pathway in HIV-1 infected individuals with neurological or psychiatric complications [182]. S-Nitrosylation of phosphatidylinositol 3-kinase (PI3K)/protein kinase B (Akt) has been demonstrated both in the brains of HIV-1 patients with HAND and in the HIV-Gp120 transgenic mouse model, leading to decreased Akt activity [208].

Another RNS-centered hypothesis for the mechanism of neuronal damage following HIV infection involves the downstream effects of nitrosative radicals produced during the immune response [209]. Proinflammatory factors (as iNOS) are released in the astrocytes by the HIV-infected macrophages [210, 211]. The severity of HIV-related dementia is correlated with the levels of iNOS expression [212]. This confirms a link between nitrosative stress and the neuroinflammatory environment in the HIV-1 infected brain [212–214].

HIV-1-infected children with high viral load exhibited higher NO blood levels than those with viral load below this threshold [215]. This and other studies in AIDS patients point at the involvement of NO in the apoptosis and functional impairment of the lymphocytes [215–217]. Pathophysiological significance of these findings was demonstrated by showing an enhanced effect of NO on HIV-1 replication* in vitro* [215]. This study has shown that the addition of NO donors together with TNF-alpha to mitogen-activated HIV-1-infected human peripheral blood mononuclear cell (PBMC) cultures produces a significant increase in viral replication, whereas the addition of iNOS specific inhibitors suppresses replication [215]. Altogether, these results suggest that NO promotes HIV-1 replication, especially in proinflammatory settings [215], which lines up with similar effects of ROS (as depicted in Section 6).

However, NO donor compounds present in the human circulatory system, such as S-nitrosothiols (RSNOs), can inhibit HIV-1 replication in acutely infected human PBMCs demonstrating an additive inhibitory effect on HIV-1 replication with 3′-azidothymidine (AZT) [218]. One of the explanatory mechanisms might be the inhibition of HIV-1 protease subjected to S-nitrosation [219, 220]. Thus, in acute HIV-1 infection, RNS may inhibit viral replication. Indeed, a study done with the fluorescent probes with an enhanced sensitivity to NO demonstrated that low NO and iNOS levels in PBMC from HIV-infected patients correlate with enhanced viral replication [221]. Interestingly, HIV-1 transgenic rats are also characterized by low NO-hemoglobin, serum nitrite, serum S-nitrosothiols, and the aortic tissue NO levels [77]. The latter indicates that the decreased levels of NO and its downstream products are linked to the direct effects of the viral proteins [77]. Their propensity to downregulate levels of RNS may create a microenvironment favouring (acute) viral infection. These considerations are in line with findings that HIV can be targeted by the compounds that affect oxidative status of the central and transitional memory T-cells: the major cellular reservoirs for HIV [222] (see Section 8 for an overview).

This set of somewhat contradictory data indicates that, in HIV-1 infection, the predominant tissue exposed to the effects of RNS is apparently the brain, while the effects of RNS on other tissues and organs of HIV-1 infected individuals may be positive and/or negative depending on RNS levels and duration of exposure.

## 8. Oxidative Stress and Antiretroviral Therapy

One of the milestone findings in the redox biology of HIV-1 was the induction of oxidative stress during antiretroviral therapy (ART). To date, numerous reports show that nucleoside and nonnucleoside RT inhibitors, as well as inhibitors of the viral protease, trigger massive ROS production in various cell types (e.g., [223–229]). Series of studies reported an increase in oxidative stress additional to the persistent redox imbalance associated with HIV-1 infection manifested by an increase in oxidants and a decrease in antioxidant serum levels [73, 230, 231]. Specifically, a study done in 84 HIV-infected patients during a 6-month period of ART demonstrated a significant increase in serum peroxidation potential, total hydroperoxide, MDA, and advanced oxidation protein product levels as well as a decrease in glutathione level, compared to their levels before the treatment and to healthy controls [232]. Ngondi et al. as well registered an aggravation of the oxidative stress by certain ART regimens in the form of a significant decrease in the levels of GSH (sulfhydryl group) [233]. Again, in patients receiving nonnucleoside reverse-transcriptase inhibitors, peroxide concentrations were significantly lower than in those treated with protease inhibitors [185]. This could be attributed to an enhancement in GSH utilization or/and to the limited intracellular reduction of its oxidized form [234]. It is generally acknowledged that the components of ART may contribute to the development of cardiovascular diseases and CNS pathologies. Some of the antiretroviral drugs, such as 2′,3′-dideoxycytidine (ddC), can penetrate the BBB and trigger oxidative stress also in the brain [235]. Experiments in the ART-exposed cell lines and laboratory animals demonstrated that the enhanced production of the oxidized metabolites occurs through the mitochondrial interference ([225]; reviewed in [73]). Mitochondrial dysfunction under ART arises from the altered replication of mitochondrial DNA and inhibited oxidative phosphorylation [236]. Some of the abovementioned dysfunctions correlate with the duration of antiretroviral therapy [237, 238]. The exact impact of oxidative stress on the efficacy of ART and HIV-1/AIDS progression and the molecular mechanisms of the redox imbalance in ART-treated HIV-infected individuals are still obscure and require further comprehensive studies.

Although ART is able to clear viremia and improve the immunological condition of HIV-infected individuals for prolonged time, the virus rebounds to levels comparable to those observed before treatment initiation shortly after treatment withdrawal due to intactness of the major cellular reservoirs for HIV, central and transitional memory T-cells (T_CM_ and T_TM_, resp.) which harbour the transcriptionally silent form of viral DNA not affected by classical antiretroviral drug regimens. Interestingly, novel oxidative stress-based therapies are arising that target these major cellular HIV reservoirs that are inaccessible to classical ART. A candidate anti-HIV reservoir compound dubbed auranofin (AF) is a prooxidant gold-based drug that inhibits thioredoxin reductases thus affecting cellular/protein redox status [239]. Auranofin was shown to exert a selective “antimemory” effect by exploiting the baseline oxidative status of lymphocytes [222]. A study by Chirullo et al. [240] explored the molecular bases of the effects of auranofin. T_CM_ and T_TM_ lymphocytes were shown to have lower baseline antioxidant defenses as compared with their naive counterparts. AF was able to exert a prodifferentiating and proapoptotic effect preferentially in these memory subsets. Namely, AF induced redox-sensitive cell death pathways initiated by an early activation of the p38 mitogen-activated protein kinase (p38 MAPK) followed by the mitochondrial depolarization and finalized by the burst in intracellular peroxides [240]. AF-induced apoptosis was inhibited by pyruvate, a well-known peroxide scavenger. Proapoptotic and prodifferentiating effects involved different pathways. Similar effect of AF was described for simian immunodeficiency virus (SIV) in monkey model [241, 242]. Additional effect on T-lymphocyte can be achieved by combining AF with drugs that inhibit glutathione biosynthesis and lower its level such as buthionine sulfoximine (BSO) [243]. Using a combination of AF, BSO, and standard ART drugs, Shytaj et al. achieved complete clearance of SIV viremia in macaques with a 100% AIDS-free survival for at least 2 years after discontinuation of the therapy [242, 244]. This data indicates that AF and other drugs inducing redox-sensitive cell death pathways can be recruited to restrict viral reservoirs* in vivo*, limit the “stem-cell-ness” of the T_CM_ and T_TM_ pools, and turn these cells into the short-lived lymphocytes [240].

## 9. Conclusions and Future Perspectives

In this review, we summarized current knowledge on the fact that HIV infection leads to a pronounced oxidative stress, described the mechanisms by which the virus triggers ROS production, and discussed the impact of HIV on antioxidant defense systems. In addition, we presented an analysis of HIV-driven oxidative stress on the associated pathology. All these data clearly show that reactive oxygen species underlie a wide spectrum of events in infected cells and tissues. At the same time, there are still notable gaps in the field that might become targets for future studies. As such, we can propose the following subjects. First, many of the multiple sources of ROS that are activated by HIV may undergo common regulation and, hence, common prohibitive or stimulative treatment. Second, the current data on the status of antioxidant defense systems are rather contradictory, and many efforts are still required to understand the actual effect of the virus in acute versus chronic infection, not only in* in vitro* and animal model systems. Third, HIV-induced oxidative stress might impact susceptibility towards other viral infections, and this question has not yet been properly addressed. Fourth, virus-triggered ROS production is a strong modulator of the immune system, a property which needs to be controlled and that can be targeted for immune suppression of viral replication. Continuation of these studies would contribute to the development of efficient antiretroviral treatments and HIV vaccines.
